# Efgartigimod for Guillain-Barré syndrome: rationale, clinical evidence, and future perspectives

**DOI:** 10.3389/fimmu.2026.1952646

**Published:** 2026-09-15

**Authors:** Xinyu Wang, Yidi Song, Wenting He, Xue Tao, Enwu Long

**Affiliations:** 1 School of Pharmacy, Southwest Medical University, Luzhou, China; 2 Department of Pharmacy, Personalized Drug Research and Therapy Key Laboratory of Sichuan Province, Sichuan Provincial People’s Hospital, University of Electronic Science and Technology of China, Chengdu, China

**Keywords:** clinical research, efficacy, efgartigimod, FcRn antagonist, Guillain-Barré syndrome, safety

## Abstract

Guillain-Barré syndrome (GBS) is an acute immune-mediated peripheral neuropathy characterized by rapidly progressive symmetric limb weakness and diminished tendon reflexes, which may be complicated by respiratory muscle paralysis in severe cases. Current first-line treatments primarily consist of intravenous immunoglobulin (IVIg) and plasma exchange (PE), but approximately 20% of patients have an incomplete response to standard therapies. Additionally, issues such as limited supply of blood products, adverse reactions, and contraindications may limit conventional treatments. Efgartigimod alfa is an engineered human IgG1 Fc fragment that targets the neonatal Fc receptor (FcRn) and competitively inhibits FcRn-mediated lgG recycling, thereby promoting the lysosomal degradation of circulating IgG, including pathogenic IgG autoantibodies, and reducing serum IgG levels. Intravenous efgartigimod alfa is approved for AChR antibody-positive generalized myasthenia gravis (gMG), whereas the U.S. approval for chronic inflammatory demyelinating polyneuropathy (CIDP) applies to the subcutaneous efgartigimod alfa/hyaluronidase co-formulation. Neither formulation is currently approved for GBS. Because IgG-mediated autoimmunity contributes to the pathogenesis of a subset of GBS, FcRn blockade provides a biologically plausible therapeutic strategy; however, its relevance may vary according to the underlying immunopathology. This narrative review summarizes the mechanistic rationale, preliminary clinical evidence, and available safety observations regarding efgartigimod in GBS, critically evaluates the limitations of the current evidence, and discusses unresolved questions and priorities for future research.

## 1 Introduction

Guillain-Barré syndrome (GBS) is an acute immune-mediated peripheral neuropathy with a global annual incidence of approximately 1–2 per 100,000 population. The disease is characterized by rapidly progressive weakness and diminished reflexes; approximately 20%–30% of patients require mechanical ventilation, and a substantial minority have persistent disability despite treatment (1, 2). GBS is not immunologically uniform. IgG antibodies against gangliosides or ganglioside complexes are pathogenic in several phenotypes, however, detectable IgG autoantibodies are absent in many patients, non-IgG antibodies may occur, and macrophage-, complement-, and T-cell-associated mechanisms may also contribute. Antibody status cannot be inferred reliably from clinical or electrophysiological subtype (3, 39). IVIg and PE remain established first-line immunotherapies with broadly comparable efficacy (4). Their limitations include incomplete response, blood-product availability, thrombotic or renal risks with IVIg in susceptible patients, and vascular access, hemodynamic, and plasma-protein losses with PE (5, 6). These limitations justify evaluation of new approaches but do not lessen the evidentiary standard required before replacing established therapy.

Efgartigimod is an FcRn antagonist that reduces circulating IgG by preventing FcRn-mediated recycling (7). Intravenous efgartigimod alfa is approved for AChR-antibody-positive generalized myasthenia gravis, whereas the United States approval for chronic inflammatory demyelinating polyneuropathy is for the subcutaneous co-formulation efgartigimod alfa and hyaluronidase-qvfc (8, 9). Neither formulation is approved for GBS. This narrative review critically evaluates the biological rationale and the low-certainty clinical evidence, emphasizing patient selection, treatment kinetics, comparison with IVIg and PE, and unresolved research priorities.

## 2 Literature search strategy

This article was designed as a narrative review. A structured literature search was conducted in PubMed/MEDLINE, Web of Science, Embase, and the Cochrane Library from database inception to July 15, 2026. ClinicalTrials.gov was additionally searched to identify ongoing or unpublished clinical trials. The search strategy combined terms related to Guillain-Barré syndrome and FcRn blockade, including (“Guillain-Barré syndrome” OR “GBS”) AND (“efgartigimod” OR “neonatal Fc receptor” OR “FcRn” OR “FcRn antagonist”). Additional searches incorporating “intravenous immunoglobulin,” “IVIg,” “plasma exchange,” “safety,” and “autoantibody” were performed when relevant. Reference lists of eligible studies and relevant reviews were also screened manually. Clinical studies reporting the administration of efgartigimod in patients with GBS were considered eligible, including randomized or non-randomized trials, retrospective or prospective cohort studies, case series, and case reports. Mechanistic studies, authoritative regulatory documents, and relevant reviews were included to contextualize the biological rationale, formulations, and approved indications of efgartigimod. Duplicate publications, studies unrelated to GBS, animal or *in vitro* studies without direct mechanistic relevance, conference abstracts lacking sufficient clinical information, and reports without extractable treatment or outcome data were excluded.

Because of the limited number and substantial heterogeneity of the available studies, no quantitative meta-analysis was performed. Evidence was categorized according to study design as comparative observational evidence, uncontrolled cohort evidence, case-series evidence, or single-case evidence. Methodological limitations were assessed qualitatively, with particular attention to sample size, baseline imbalance, non-random treatment selection, confounding by indication, outcome heterogeneity, missing data, spontaneous recovery, and publication bias. As this was a narrative rather than a systematic review, the possibility of incomplete retrieval and selective citation cannot be excluded.

## 3 Theoretical basis

The biological rationale for applying efgartigimod to GBS is based on the contribution of pathogenic IgG autoantibodies to peripheral nerve injury in a subset of patients. However, GBS is immunopathologically heterogeneous, and IgG-mediated mechanisms do not account for all cases. The contribution of humoral immunity varies across GBS phenotypes. In axonal forms such as acute motor axonal neuropathy (AMAN), IgG anti-ganglioside antibodies, including anti-GM1 and anti-GD1a antibodies, can contribute to complement-mediated nodal and axonal injury, whereas the immunopathology of acute inflammatory demyelinating polyneuropathy (AIDP) is more heterogeneous and may involve humoral, complement-dependent, macrophage-mediated, and cellular immune mechanisms (3, 39). Moreover, IgM anti-ganglioside antibodies have also been identified in some patients with GBS, indicating that not all antibody-mediated disease is necessarily IgG dependent. Importantly, electrophysiological subtype does not correspond one-to-one with antibody status or immunoglobulin isotype and therefore cannot reliably serve as a surrogate for identifying an IgG-mediated mechanism. In some patients with acute motor neuropathy, pathogenic anti-GM1/GA1 antibodies are predominantly of the IgG1 and IgG3 subclasses, both of which have strong complement-fixing capacity (40). These observations provide a mechanistic basis for targeting IgG in selected patients, while also indicating that the relevance of FcRn blockade may vary according to the underlying immunopathology. The neonatal Fc receptor (FcRn) is widely expressed and protects IgG from lysosomal degradation by recycling internalized IgG back to the circulation, thereby contributing to the prolonged serum half-life of IgG. FcRn binding may vary according to IgG subclass and allotype (41). Efgartigimod is an engineered human IgG1-derived Fc fragment with increased affinity for FcRn. By competitively inhibiting FcRn-mediated IgG recycling, efgartigimod promotes lysosomal degradation of circulating IgG and reduces total IgG levels, including pathogenic IgG autoantibodies (**Figure 1**) (7, 10, 20). Importantly, this mechanism is not antigen specific and does not distinguish pathogenic IgG from non-pathogenic or therapeutic IgG. In addition, FcRn blockade does not directly reduce other immunoglobulin classes such as IgM or IgA, which may limit its biological relevance in patients whose disease is predominantly driven by non-IgG-mediated mechanisms. At present, there is insufficient evidence to recommend routine empiric FcRn blockade before the relevant antibody status is known, and it also remains uncertain whether treatment should be systematically deferred until an IgG-mediated mechanism has been confirmed. Prospective studies are required to define the optimal patient-selection strategy. The established effects of efgartigimod in other IgG-mediated neurological diseases provide proof of principle for FcRn blockade but should not be regarded as direct evidence of efficacy in GBS. In the phase III ADAPT trial in acetylcholine receptor (AChR) antibody-positive generalized myasthenia gravis (gMG), efgartigimod reduced total IgG and AChR autoantibody levels, and 68% of AChR antibody-positive patients achieved a Myasthenia Gravis Activities of Daily Living (MG-ADL) response, with an acceptable safety profile (8). In addition, the subcutaneous efgartigimod alfa/hyaluronidase co-formulation has been approved in the United States for chronic inflammatory demyelinating polyneuropathy (CIDP) (9).

**Figure 1:**
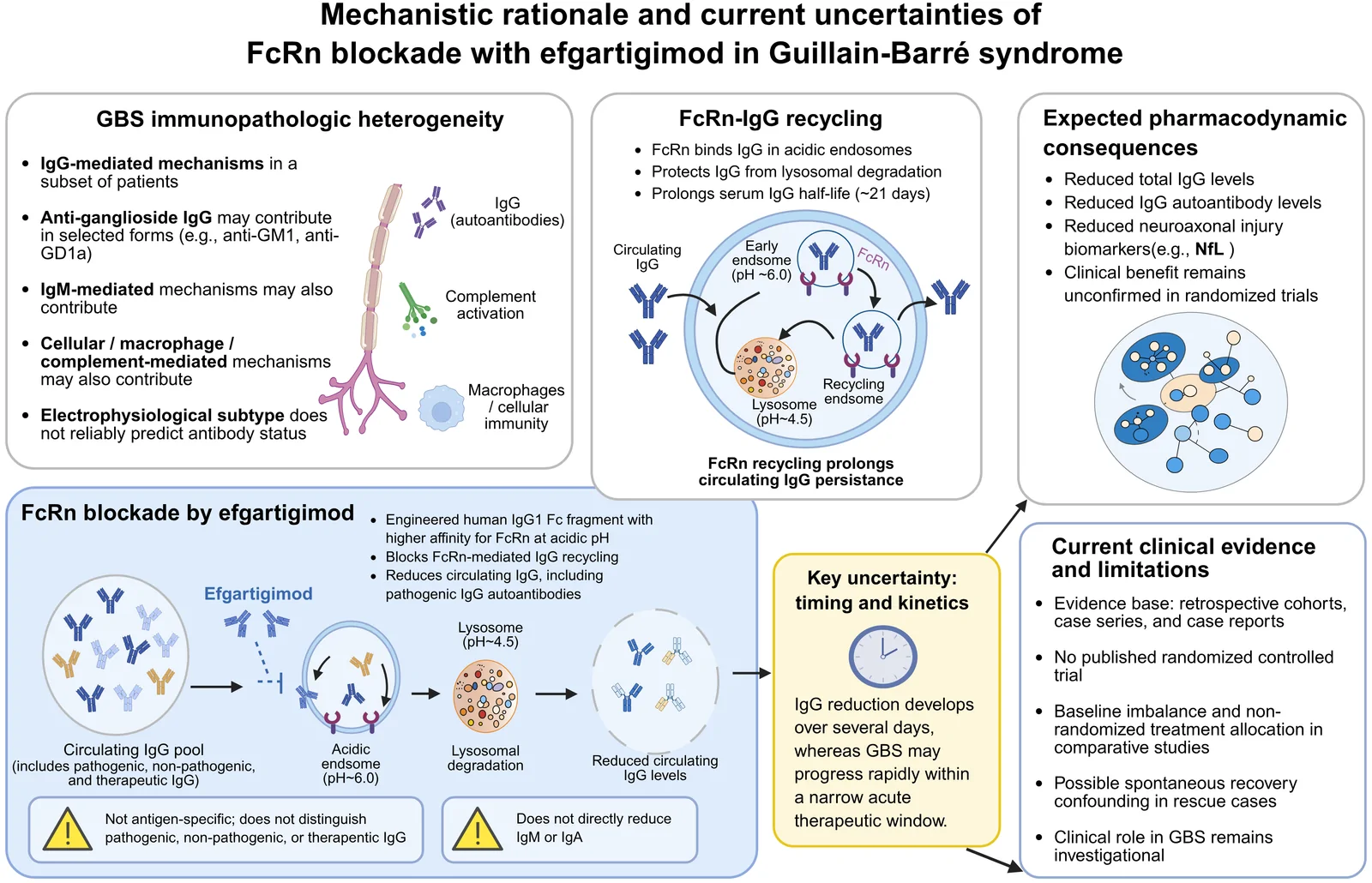
Mechanistic rationale and current uncertainties of FcRn blockade with efgartigimod in Guillain-Barré syndrome. Guillain-Barré syndrome (GBS) is immunopathologically heterogeneous, with IgG-mediated mechanisms contributing to disease in only a subset of patients, while IgM antibodies and cellular, macrophage, and complement-mediated mechanisms may also be involved. FcRn normally protects IgG from lysosomal degradation by recycling IgG back to the circulation. Efgartigimod competitively inhibits FcRn-mediated IgG recycling, thereby increasing lysosomal degradation of circulating IgG, including pathogenic IgG autoantibodies. This effect is not antigen-specific and does not distinguish pathogenic IgG from non-pathogenic or therapeutic IgG; FcRn blockade also does not directly reduce IgM or IgA. Although reductions in total IgG, IgG autoantibodies, and neuroaxonal injury biomarkers may occur, clinical benefit in GBS remains unconfirmed. In addition, IgG reduction develops progressively over several days, which may not fully align with the narrow acute therapeutic window of rapidly progressive GBS. Current evidence is derived mainly from retrospective observational studies, case series, and case reports, and the clinical role of efgartigimod in GBS remains investigational. FcRn, neonatal Fc receptor; GBS, Guillain-Barré syndrome; IgG, immunoglobulin G; IgM, immunoglobulin M; IgA, immunoglobulin A; NfL, neurofilament light chain.

Although CIDP and GBS are both immune-mediated peripheral neuropathies, they differ substantially in disease course and immunopathogenesis. Therefore, experience with FcRn blockade in gMG and CIDP supports the biological plausibility of IgG lowering in peripheral immune-mediated neurological disease but cannot be directly extrapolated to establish therapeutic efficacy in acute GBS.

An important limitation of this mechanistic rationale is the potential mismatch between the pharmacodynamic kinetics of FcRn blockade and the acute therapeutic window of GBS. Efgartigimod reduces circulating IgG progressively over several days rather than producing immediate antibody removal, whereas GBS may progress rapidly and typically reaches its clinical nadir within approximately 2–4 weeks. Irreversible nodal or axonal injury may therefore occur before maximal IgG reduction is achieved. Consequently, although FcRn blockade is biologically plausible, it remains uncertain whether its onset of action is sufficiently rapid to provide clinically meaningful benefit as first-line monotherapy in patients with rapidly progressive or severe GBS. The temporal relationship between IgG reduction, disease stabilization, and neurological recovery should therefore be specifically evaluated in prospective studies.

Available observational studies have reported pharmacodynamic and biomarker changes that are consistent with the expected effects of FcRn blockade in GBS. Bi et al. (11) reported a mean reduction in total IgG of 67.3 ± 8.2% after treatment in patients receiving efgartigimod as first-line therapy, consistent with the expected pharmacodynamic effect of FcRn antagonism. Zhang et al. (12) reported that neurofilament light chain (NfL) levels decreased by 58.4% from baseline at week 2 in the efgartigimod group compared with 32.1% in the IVIg group (P = 0.01). Although this finding may be consistent with reduced ongoing neuroaxonal injury, the observational design, small sample size, and baseline differences between treatment groups preclude causal attribution to efgartigimod or conclusions that FcRn blockade terminates axonal injury earlier than IVIg. The same study reported a greater reduction in anti-GM1 antibody titers in the efgartigimod group than in the IVIg group (71.2% vs. 43.5%, P = 0.008). This observation is consistent with the expected reduction of circulating IgG autoantibodies during FcRn blockade; however, because efgartigimod is not antigen specific, it should not be interpreted as evidence of selective or targeted clearance of anti-GM1 antibodies. Taken together, these biomarker findings support the expected pharmacodynamic activity of FcRn blockade but do not establish clinical efficacy. Whether the magnitude or timing of reductions in total IgG, pathogenic autoantibodies, or NfL translates into clinically meaningful neurological recovery or improved long-term outcomes remains to be determined in adequately powered prospective randomized studies.

## 4 Clinical research evidence

Current clinical evidence for efgartigimod in GBS remains limited and is derived primarily from retrospective cohort studies, observational studies, case series, and case reports. These studies have explored several clinical scenarios, including efgartigimod as first-line therapy, rescue therapy after inadequate response to IVIg or PE, comparative observational analyses with conventional treatments, and reports involving refractory cases or specific GBS phenotypes. However, the overall certainty of evidence remains low because available studies are limited by small sample sizes, non-randomized treatment allocation, baseline heterogeneity, and potential confounding from the natural recovery course of GBS. Therefore, current findings should be interpreted as preliminary clinical signals and hypothesis-generating observations rather than definitive evidence of efficacy or comparative effectiveness.

### 4.1 Efgartigimod as first-line therapy for GBS: preliminary evidence

Bi et al. (11) conducted a single-center retrospective study in China involving 36 GBS patients, of whom 17 received efgartigimod as first-line therapy. In this cohort, the median time to independent walking in the first-line treatment group was 6 days; at week 1, the GBS disability scale (GBS-DS) score improved by 2.0 points, and the Medical Research Council (MRC) sum score improved by 9.0 points; total IgG decreased by a mean of (67.3 ± 8.2)% after treatment. Regarding safety, only 2 patients (5.6%) reported mild adverse events (1 case of rash, 1 case of headache), with no serious adverse events reported. Although these findings demonstrate the expected pharmacodynamic effect of FcRn blockade and suggest a favorable clinical trajectory, the lack of a randomized comparator limits the ability to determine whether the observed recovery exceeded the expected natural course of GBS. Liu et al. (13) conducted a Chinese retrospective study involving 34 GBS patients who were unable to walk independently, with 12 patients receiving efgartigimod as first-line therapy. The 4-week independent walking rate was 100% (12/12), and the 12-week independent running capacity recovery rate was 100% (12/12). Exploratory subgroup analysis described favorable outcomes among patients with ophthalmoplegia or respiratory insufficiency; however, the extremely small subgroup sizes, absence of interaction testing, and non-random treatment allocation preclude conclusions regarding differential treatment response.

Overall, these studies provide preliminary clinical signals that efgartigimod may have therapeutic potential as first-line therapy for selected patients with GBS. However, both studies were retrospective and included small efgartigimod-treated groups (17 and 12 patients, respectively). Non-randomized treatment allocation, baseline heterogeneity, and the absence of controlled comparisons limit causal inference. Therefore, whether efgartigimod accelerates neurological recovery or provides advantages over established first-line therapies remains to be determined in prospective randomized studies.

### 4.2 Efgartigimod as rescue therapy for IVIg/PE-refractory GBS: preliminary evidence

Therapeutic options remain limited for patients with GBS who show inadequate improvement after IVIg or PE, representing an important unmet clinical need. Available evidence regarding efgartigimod as rescue therapy is currently derived mainly from small retrospective cohorts and case reports. In the study by Bi et al. (11), 19 patients who were unresponsive to IVIg/PE received efgartigimod as rescue therapy, with a median time to independent walking of 25 days. Although neurological improvement was observed after treatment, the absence of a matched control group limits the ability to determine whether recovery exceeded the expected natural course of GBS. Zhang et al. (14) reported 5 patients with poor response to IVIg; following efgartigimod administration, four patients showed clinical improvement within 2 weeks, with a mean increase in MRC sum score of 12.5 points. However, the small sample size and uncontrolled design limit causal interpretation. Ripellino et al. (15) reported a 60-year-old female patient with AIDP-type GBS who progressed to mechanical ventilation despite PE and IVIg. Following initiation of efgartigimod at week 4 after disease onset, voluntary limb movement returned on day 7, ventilator weaning occurred on day 14, and independent walking was achieved at 4 months. Although clinically encouraging, this observation cannot exclude spontaneous neurological recovery occurring during the plateau-to-recovery transition phase of GBS. Hu et al. (16) described three patients with poor IVIg response who showed reductions in GBS-DS scores after efgartigimod treatment, with a median onset time of 5 days and no adverse events. These reports provide preliminary signals that efgartigimod may have potential as a rescue strategy in selected patients with refractory GBS; however, the current evidence remains insufficient to establish causal efficacy.

This limitation is particularly important because GBS is typically a monophasic disorder. A multicenter prospective cohort study involving 297 GBS patients demonstrated that 98% achieved symptom stabilization within 4 weeks after onset and 85% showed improvement during the same period, followed by a variable recovery phase with an average clinical recovery duration of approximately 200 days (17). In many reported rescue cases, efgartigimod was initiated around week 4 after onset, coinciding with the transition from disease plateau to spontaneous recovery. Therefore, observed neurological improvement cannot be definitively attributed to pharmacological effects of efgartigimod rather than the natural disease course. Prospective controlled studies, including randomized designs or carefully matched observational studies, are required to clarify the true efficacy of efgartigimod in IVIg/PE-refractory GBS.

### 4.3 Comparison with other therapeutic modalities

#### 4.3.1 Comparison with intravenous immunoglobulin

Several retrospective and observational studies have compared efgartigimod with IVIg; however, the non-randomized designs, small sample sizes, and baseline differences between treatment groups limit the validity of direct comparative conclusions. Liu et al. (13) reported a 4-week independent walking rate of 100% in the efgartigimod group vs. 64% in the IVIg group (P = 0.03), and a 12-week independent running recovery rate of 100% vs. 55% (P = 0.006). Although these numerical differences favored the efgartigimod group, the retrospective and non-randomized design prevents attribution of the observed differences to treatment effect alone. In the two-center retrospective study by Zhang et al. (12), the primary clinical endpoint, GBS-DS score ≤2 at week 4, was not significantly different between groups (OR = 0.67, 95% CI [0.10, 4.48]; P = 1.000). A greater improvement in MRC sum score was observed in the efgartigimod group (P = 0.007); however, the efgartigimod group had less severe baseline impairment, including a higher baseline MRC sum score, which may have favored subsequent outcomes. Cai et al. (18) reported a median time to improvement of 4 days in the efgartigimod group and 8 days in the IVIg group, with week-2 GBS-DS response rates of 90.9% and 81.8%, respectively. Because treatment allocation was non-randomized and the sample size was small, these observations should not be interpreted as evidence of a faster clinical response with efgartigimod. Biomarker analyses also showed greater reductions in NfL (58.4% vs. 32.1%, P = 0.01) and anti-GM1 antibody levels (71.2% vs. 43.5%, P = 0.008) in the efgartigimod group. These findings are consistent with pharmacodynamic activity but do not establish superior clinical efficacy. Adverse-event rates were broadly similar between groups, and no thrombotic or renal impairment events were reported in the efgartigimod groups; however, the available sample sizes are insufficient to establish a comparative safety advantage. Overall, current observational evidence does not establish superiority, non-inferiority, or a faster response of efgartigimod compared with IVIg. Prospective randomized controlled trials with balanced baseline characteristics are required to determine whether efgartigimod provides clinically meaningful comparative benefit.

Tao et al. (30) reported a dual-center retrospective cohort of 52 patients treated with IVIg (n = 20), efgartigimod (n = 16), or IVIg followed by efgartigimod (ISE; n = 16). A reduction of at least 2 points in GBS-DS was more frequent in the efgartigimod group than in the IVIg group at week 2 (37.5% vs. 0%; P < 0.01) and at the final follow-up (81.3% vs. 40.0%; P = 0.02). However, the proportion attaining GBS-DS ≤1 did not differ significantly among groups at any scheduled visit, and the mean time to reach GBS-DS ≤1 was also not significantly different (2.6 ± 1.2 weeks with efgartigimod, 3.1 ± 1.1 weeks with IVIg, and 2.8 ± 1.2 weeks with ISE; P = 0.62). Treatment-emergent adverse events were recorded in 3 of 16 efgartigimod-treated patients, including two venous thromboses and one electrolyte imbalance. Because treatment allocation was non-random, efgartigimod use was influenced by clinical factors such as IVIg intolerance, contraindications, patient preference, or availability, and the ISE group was selected after an inadequate response to IVIg. Accordingly, these findings are susceptible to confounding by indication and do not establish comparative efficacy, faster recovery, or a safety advantage.

#### 4.3.2 Comparison with plasma exchange

PE removes pathogenic antibodies through physical means, with rapid onset but requiring vascular access and special equipment, and can lead to loss of coagulation factors and albumin. Efgartigimod reduces circulating IgG by inhibiting FcRn-mediated recycling, with convenient administration (once-weekly intravenous infusion) and no consumption of albumin or coagulation factors. Currently, no randomized head-to-head trial comparing efgartigimod with PE is available. Cheng et al. (19) compared the two groups in a retrospective controlled study, with a median time to 1-point improvement in GBS-DS of 4 days in the efgartigimod group (n = 17) vs. 11.5 days in the PE group (n = 4) (P = 0.033). At week 1 of treatment, 80% (4/5) of patients in the efgartigimod group achieved an Inflammatory Neuropathy Cause and Treatment (INCAT) disability scale score ≤ 2, compared to 25% (1/4) in the PE group. It should be noted that the PE group included only 4 patients, an extremely small sample size, rendering this comparison exploratory only; these findings cannot establish superiority, non-inferiority, or a faster clinical response of efgartigimod compared with PE. However, PE also depletes beneficial components such as albumin and fibrinogen, requires vascular access, and carries risks of infection, bleeding, and hypotension. Efgartigimod, as an FcRn antagonist that enhances IgG catabolism, does not deplete other plasma proteins and may offer practical advantages in administration; however, its onset depends on sustained FcRn inhibition *in vivo*, and the clearance efficiency shows considerable inter-individual variability. For severe GBS patients with concomitant coagulation disorders or hypoalbuminemia, the use of PE may carry risks of increased bleeding or further plasma protein loss. In contrast, efgartigimod does not deplete coagulation factors or albumin; however, these differences represent mechanistic and practical considerations rather than evidence of superior clinical efficacy. However, it should be noted that efgartigimod has inherent limitations: first, its pharmacodynamic effect depends on sustained FcRn inhibition, and substantial reductions in IgG levels typically require several days, which may not fully align with the narrow acute therapeutic window of rapidly progressive GBS; second, FcRn blockade primarily lowers circulating IgG, whereas its effects on antibodies already bound within neural tissues are less clearly established; third, FcRn blockade reduces circulating IgG but does not directly reduce other immunoglobulin classes such as IgM or IgA (20). Given that some patients with GBS may harbor IgM-type anti-ganglioside antibodies (21), the benefit of efgartigimod may therefore be limited or uncertain in patients whose disease is predominantly mediated by non-IgG mechanisms. Therefore, future patient selection for FcRn blockade may require consideration of individual serological and immunopathological profiles.

#### 4.3.3 Comparison with other emerging biologics

Eculizumab: Complement activation plays an important role in nerve injury in GBS. An early phase 2 trial suggested that eculizumab appeared safe in GBS patients (22). However, in the phase 3 trial, the primary endpoint was the time to first achieve a Hughes functional grade score ≤1; this endpoint was not met (HR = 0.90, 95% CI 0.45–1.97; P = 0.89) (23). Although endpoint selection and treatment timing may influence the detection of therapeutic effects in acute GBS, these considerations do not alter the fact that the phase 3 trial failed to demonstrate efficacy on its prespecified primary endpoint. Several reviews have discussed FcRn inhibition and complement inhibition as emerging therapeutic strategies under investigation in GBS (24–26). However, as a monoclonal antibody, eculizumab exerts its effect by inhibiting the C5 protein downstream of the complement cascade, differing from the mechanism of efgartigimod, which reduces circulating IgG through FcRn blockade.

Rituximab: Evidence regarding the use of rituximab in GBS remains very limited. To date, only a few case reports have documented its application in refractory GBS, including one patient who showed clinical improvement following low-dose rituximab after unsuccessful treatment with both IVIg and plasma exchange (27), and another patient who developed GBS after immune checkpoint inhibitor therapy and showed clinical improvement following rituximab treatment (28). Rituximab has also been evaluated in other immune-mediated peripheral neuropathies such as CIDP, but such evidence cannot be directly extrapolated to acute GBS (29).

Immunosuppressants: Conventional immunosuppressants (such as corticosteroids) are not routinely recommended in the acute phase of GBS. Multiple clinical trials have shown that corticosteroid monotherapy does not improve outcomes in GBS (31). This lack of efficacy highlights the need to investigate alternative immunomodulatory strategies with mechanisms more closely related to the specific immune pathways involved in GBS.

### 4.4 Efgartigimod in specific subtypes and special populations

#### 4.4.1 Specific GBS subtypes

Fan et al. (32)reported 2 patients with Miller-Fisher syndrome (MFS)/GBS overlap syndrome (anti-GQ1b antibody-positive) who had limited response to IVIg combined with corticosteroids; after adding efgartigimod, ophthalmoplegia and limb weakness gradually improved, and anti-GQ1b antibody titers decreased substantially. Zhou et al. (33)reported 1 patient with AMAN who was unresponsive to IVIg; after switching to efgartigimod, muscle strength improved, with only mild foot drop remaining at long-term follow-up. These case reports provide preliminary observations of clinical improvement in selected antibody-positive GBS phenotypes; however, the very small number of reported cases precludes conclusions regarding efficacy across GBS subtypes. Moreover, electrophysiological subtype does not correspond one-to-one with antibody status or immunoglobulin isotype; therefore, future studies should evaluate responses according to both GBS phenotype and serological characteristics rather than subtype alone.

#### 4.4.2 Patients with IVIg contraindications

Deng et al. (34) reported a 47-year-old female patient with AMAN-type GBS who had a clear contraindication to IVIg due to a history of IVIg-related thrombosis. Efgartigimod was selected as first-line therapy, with clinical improvement observed on day 4 of treatment and no adverse events throughout the course. This case suggests that efgartigimod may warrant further investigation as a potential option for selected patients in whom IVIg is contraindicated; however, a single case report cannot establish its efficacy or safety as an alternative first-line therapy.

#### 4.4.3 Drug-induced GBS

Xie et al. (35) reported a patient with levofloxacin-induced AMAN who had received multiple sessions of PE, corticosteroid pulse therapy, and IVIg, but still had severe residual limb weakness. After switching to efgartigimod, muscle strength and activity gradually improved. This report documents the use of efgartigimod in levofloxacin-associated GBS; however, a single uncontrolled case cannot establish whether drug-induced GBS represents a population that is particularly responsive to FcRn blockade.

#### 4.4.4 Sequential efgartigimod and IVIg therapy

Chen et al. (36) administered efgartigimod sequentially after IVIg (efgartigimod immediately following IVIg) in 4 patients. Compared with historical controls, the median hospital stay decreased from 21 days to 12 days (P = 0.03), and the median duration of mechanical ventilation decreased from 12 days to 6 days (P = 0.04). However, the case series included only four patients and used historical controls, substantially limiting causal and comparative inference. Tao et al. (30) additionally described 16 patients treated with IVIg followed by efgartigimod after an inadequate response to IVIg; outcomes in this selected group were descriptive and cannot be separated from indication bias, prior IVIg exposure, or spontaneous recovery. Although IVIg and efgartigimod act through different immunomodulatory mechanisms, their sequential use cannot currently be assumed to be additive or synergistic. Because FcRn blockade does not distinguish pathogenic endogenous IgG from therapeutic IgG administered as IVIg, efgartigimod given immediately after IVIg may accelerate the clearance of infused immunoglobulin and potentially reduce its circulating exposure. The clinical significance of this interaction and the optimal interval between IVIg and efgartigimod remain unknown.

Taken together, currently available retrospective studies, observational cohorts, and case reports provide preliminary signals of neurological improvement following efgartigimod treatment in GBS. However, the small sample sizes, non-randomized treatment allocation, baseline imbalance, and potential confounding by spontaneous recovery prevent firm conclusions regarding clinical efficacy or comparative speed of response. Reported outcomes in individual studies included a median time to independent walking of 6 days in a first-line cohort, a week-2 GBS-DS improvement rate exceeding 90% in one observational study, and a 4-week independent walking rate of 100% in a small retrospective cohort. These estimates should be interpreted in the context of the methodological limitations of the underlying studies. Biomarker studies have reported reductions in NfL and anti-ganglioside antibody levels following efgartigimod treatment. These findings are consistent with pharmacodynamic activity but do not constitute direct evidence of antigen-specific antibody clearance or establish clinical efficacy. Across the available published cohorts and case reports, accumulated safety experience remains limited, with predominantly mild adverse events reported to date. However, the available sample size and follow-up duration remain insufficient to reliably detect uncommon or rare adverse events. A summary and critical appraisal of the currently available clinical evidence is provided in **Table 1**.

**Table 1:** Summary and critical appraisal of clinical evidence for efgartigimod in Guillain-Barré syndrome.

Study	Design/sample	Reported signal	Key limitations/bias	Evidence interpretation
Bi et al. (11)	Single-center retrospective; 36 total (17 first-line, 19 rescue)	Median time to independent walking 6 days (first-line) and 25 days (rescue); marked total IgG reduction; few mild AEs	No randomized comparator; small groups; rescue timing overlaps with spontaneous recovery; treatment-selection bias	Pharmacodynamic activity and exploratory clinical signal; causal efficacy not established.
Cai et al. (18)	Multicenter observational; 22 total	Median improvement time reported as 4 vs 8 days; week-2 response 90.9% vs 81.8%; similar overall AE frequency	Small sample; non-randomized allocation; residual confounding; limited power for comparative inference	Cannot establish faster response or non-inferiority/superiority vs IVIg
Zhang et al. (12)	Two-center retrospective; 21 total	Greater MRC improvement; larger NfL and anti-GM1 reductions; primary week-4 GBS-DS endpoint not significantly different	Baseline imbalance including higher MRC in efgartigimod group; small sample; non-randomized treatment	Biomarker signal is hypothesis-generating; clinical superiority not established
Cheng et al. (19)	Retrospective controlled; efgartigimod n=17; PE n=4	Shorter reported time to 1-point GBS-DS improvement	PE comparator n=4; unstable estimates; strong risk of selection/confounding; no adequate head-to-head evidence	Exploratory only; no basis to prefer efgartigimod over PE
Liu et al. (13)	Retrospective cohort; 34 total; efgartigimod n=12	Higher reported week-4 independent walking and week-12 running recovery rates	Small treated group; non-randomized treatment selection; potential baseline and indication bias	Preliminary association; requires randomized confirmation
Tao et al. (30)	Dual-center retrospective; 52 total (IVIg 20, efgartigimod 16, IVIg→efgartigimod 16)	≥2-point GBS-DS improvement favored efgartigimod at week 2 and final follow-up; time to GBS-DS ≤1 was not significantly different; 3/16 TEAEs	Non-random allocation; confounding by indication; variable dosing; small groups; sequential group selected after inadequate IVIg response	Comparative observational signal only; no proof of superiority, faster recovery, or safety advantage

All current GBS evidence is observational or case-based should therefore be regarded as low-certainty evidence. The “evidence interpretation” column is a qualitative critical appraisal based on study design, sample size, baseline comparability, and confounding risk; it is not a formal GRADE assessment. AE, adverse event; GBS-DS, Guillain-Barré syndrome disability scale; IVIg, intravenous immunoglobulin; MRC, Medical Research Council sum score; NfL, neurofilament light chain; PE, plasma exchange.

## 5 Limitations and future perspectives

All studies included in this review are retrospective observational studies or case reports, with no prospective randomized controlled trials published to date, resulting in a generally low level of evidence. In addition, because this is a narrative rather than a systematic review, study identification and selection were not based on a prespecified systematic-review protocol, and selective-citation bias cannot be completely excluded.Retrospective studies are inherently subject to selection bias, information bias, and inadequate control for confounders, while case reports are limited by publication bias (positive results are more likely to be published) and small sample sizes. Furthermore, in the salvage therapy setting, the timing of efgartigimod administration coincides with the natural recovery phase of GBS, making it difficult to distinguish drug effects from spontaneous remission based on existing case reports, thereby introducing uncertainty into causal attribution. Another important uncertainty is the potential mismatch between the pharmacodynamic kinetics of FcRn blockade and the acute therapeutic window of GBS. Substantial IgG reduction develops progressively over several days, whereas GBS may progress rapidly and typically reaches its clinical nadir within 2–4 weeks. Therefore, it remains uncertain whether FcRn blockade can reduce pathogenic IgG sufficiently early to prevent ongoing nodal or axonal injury in rapidly progressive disease. The included studies also exhibit considerable heterogeneity in dosing regimens, outcome measures, and evaluation time points, precluding direct pooled analyses. Regarding safety, accumulated experience remains limited. Although most earlier reports described mild events, Tao et al. (30) recorded two venous thromboses and one electrolyte imbalance among 16 efgartigimod-treated patients. These events cannot be causally attributed in a retrospective cohort, but they reinforce the need for systematic prospective safety monitoring, particularly for thrombotic and infectious complications. In terms of patient coverage, all current evidence derives from off-label use. While one case report has described the use of efgartigimod in a pregnant patient with myasthenia gravis (37), no such data are available for pregnant patients with GBS; data for other special populations, including children, elderly patients, and those with hepatic or renal impairment, are also lacking.

Two interventional studies of efgartigimod in GBS have been registered. NCT05701189, a planned randomized phase 2 trial, was withdrawn because of recruitment difficulties without enrolling participants, whereas NCT06885762 is registered as an open-label, single-group phase 2/3 study with a small planned sample size. Therefore, adequately powered randomized controlled evidence for efgartigimod in GBS remains unavailable, and future controlled trials are still needed to establish its efficacy, safety, optimal timing, and clinical role. In addition, several directions warrant further exploration: whether responses to efgartigimod differ according to antibody status, immunoglobulin isotype, autoantibody specificity, and GBS phenotype (e.g., AIDP, AMAN, or MFS); the pharmacokinetic and clinical consequences of sequential or combination treatment with IVIg, including whether FcRn blockade accelerates the clearance of infused therapeutic IgG and the optimal interval between treatments; the feasibility and implications of intravenous versus subcutaneous administration in acutely ill patients, particularly those requiring intensive care or mechanical ventilation; and the safety, efficacy, and cost-effectiveness of efgartigimod in pediatric patients with GBS.

## 6 Conclusion

Efgartigimod, as a novel FcRn antagonist, has shown preliminary pharmacodynamic and clinical signals in GBS; however, the current evidence remains limited and of low certainty. Available observational evidence suggests possible neurological improvement following treatment, but it does not establish that efgartigimod accelerates recovery or has a faster onset of action than IVIg or PE. Its potential role as rescue therapy after an inadequate response to standard treatment also remains uncertain.FcRn antagonism provides a mechanistically distinct strategy for reducing circulating IgG, including pathogenic IgG autoantibodies, and represents an emerging area of investigation in GBS (38). At present, the evidence is insufficient to define efgartigimod as an established alternative to IVIg or PE, including in patients with inadequate response, intolerance, or contraindications to standard therapy. Because efgartigimod is not approved for GBS, any current use remains off-label and should follow relevant institutional procedures with appropriate informed consent and multidisciplinary clinical oversight. However, the current evidence base is of very low certainty and is derived predominantly from small retrospective studies, case series, and case reports, with substantial risks of selection bias, confounding, and spontaneous-recovery bias. Although interventional studies of efgartigimod in GBS have been registered, adequately powered randomized controlled evidence remains unavailable. Future prospective controlled trials are required to determine its efficacy, safety, optimal timing, and potential clinical role in GBS. Until higher-quality prospective evidence becomes available, efgartigimod should be regarded as an investigational or off-label strategy rather than an established therapeutic option for GBS.
